# Generation of disease-specific induced pluripotent stem cells from patients with different karyotypes of Down syndrome

**DOI:** 10.1186/scrt105

**Published:** 2012-04-18

**Authors:** Xiaoning Mou, Yuanbo Wu, Henghua Cao, Qingzhang Meng, Qihui Wang, Chengchao Sun, Shengshou Hu, Yue Ma, Hao Zhang

**Affiliations:** 1 National Laboratory of Biomacromolecules, Institute of Biophysics, Chinese Academy of Sciences, 15 Datun Road, Beijing 100101, China; 2 Graduate University of the Chinese Academy of Sciences, 19 Yuquan Road, Beijing 100049, China; 3 Key Laboratory for Cardiac Regeneration Medicine, Ministry of Health, Fuwai Hospital, 167 Belishi Road, Beijing 100037, China; 4 State Key Laboratory of Cardiovascular Disease, Fuwai Hospital, National Center for Cardiovascular Diseases, Chinese Academy of Medical Sciences and Peking Union Medical College, 167 Belishi Road, Beijing 100037, China; 5 Department of Cardiac Surgery, Fuwai Hospital, Chinese Academy of Medical Sciences, 167 Belishi Road, Beijing 100037, China; 6 Department of Thoracic and Cardiovascular Surgery, First Affiliated Hospital of Wenzhou Medical College, 2 Fuxue Lane, Wenzhou 325000, China

## Abstract

**Introduction:**

Down syndrome (DS), a major cause of mental retardation, is caused by trisomy of some or all of human chromosome 21 and includes three basic karyotypes: trisomy 21, translocation, and mosaicism. The derivation of DS-specific induced pluripotent stem cells (iPSCs) provides us novel DS models that can be used to determine the DS mechanism and to devise therapeutic approaches for DS patients.

**Methods:**

In the present study, fibroblasts from patients with DS of various karyotypes were reprogrammed into iPSCs via the overexpression of four factors: OCT4, SOX2, KLF4, and c-MYC, by using lentiviral vectors. The abilities of the iPSC-DS in the self-renewal and pluripotency *in vitro *and *in vivo *were then examined.

**Results:**

The iPSC-DS showed characteristics similar to those of human embryonic stem cells, particularly the morphology, surface marker (SSEA4, TRA-1-60, and TRA-1-81) expression, pluripotent-specific transcription-factor expression levels, and methylation status of the OCT4 promoter. The pluripotency of iPSC-DS was also tested *in vitro *and *in vivo*. Embryoid bodies were formed and showed the expression of differentiated markers for three germ layers. Furthermore, iPSC-DS formed classic teratomas when injected into nonobese diabetic-severe combined immunodeficient (NOD-SCID) mice.

**Conclusions:**

iPSCs were generated from patients with DS. The iPSCs derived from different types of DS may be used in DS modeling, patient-care optimization, drug discovery, and eventually, autologous cell-replacement therapies.

## Introduction

Down syndrome (DS) is the major cause of congenital heart disease and the most frequent genetic cause of mental retardation, which occurs in roughly one of 700 live births [1]. In addition to the characteristic dysmorphology of the facial and physical features, DS is associated with increased risks of leukemia, immune system defects, and an early Alzheimer-like dementia [2]. DS includes caused by the presence of an extra chromosome 21 (trisomy 21), subsets of the phenotypic features of DS may be caused by the duplication of parts of the chromosome 21(translocation) [2,3]. Although translocation affects only about four of 100 people with DS, it is also associated with a number of deleterious phenotypes. Mosaicism results from the abnormal cell division in some cells after fertilization, with some of the cells having 47 chromosomes, and the others being normal [4,5]. Understanding and investigating the mechanisms of DS present a considerable challenge because of the genetic complexity and individual variability of the DS phenotypes.

An important approach to the genetic analysis of DS is the establishment and study of analogous mouse models. Although several types of DS mouse models, such as Ts65Dn [6,7] and Ts1Cje [8,9], are useful, the mouse model should have a phenotype that can be reasonably related to the human condition. Human embryonic stem cells (hESCs) have proven to be another source for the modeling of genetic diseases [10]; however, the establishment of hESCs with DS is limited by ethical dilemmas. In addition, the transplantation of hESCs-differentiated cells can trigger an immune rejection by the host. Induced pluripotent stem cells (iPSCs) possess the properties of hESCs, such as self-renewal and pluripotent potentials, but are superior to hESCs in the generation of disease-specific and patient-specific iPSCs without any accompanying controversy [11-14]. Recent studies showed that a variety of disease-specific iPSCs may be successfully produced [15-22]. Even in mouse and rat models, disease phenotypes were significantly ameliorated by gene-corrected, iPSC-differentiated cells [23-25]; these results provide the theoretic basis for iPSC-based therapies.

In the current study, iPSCs were generated from DS patients with different karyotypes (trisomy 21 and translocation) via the overexpression of four factors: OCT4, SOX2, KLF4, and c-MYC. The generated DS-specific iPSCs can be effective cell models for the study of the DS mechanism as well as for drug screening. Furthermore, the iPSCs can be alternative cell sources for autologous cell-replacement therapies.

## Materials and methods

### Ethics statement

All animal experimental procedures and protocols were approved by the Ethics Committee of Fu Wai Hospital and were in accordance with the Guide for the Care and Use of Laboratory Animals (approval number: 0000284). Written approvals for human skin-tissue collection, subsequent iPSC generation, and genome/gene analyses performed in the current study were obtained from the Ethics Committee for Human Research at Fu Wai Hospital (approval number: 241). Two patients are involved in the study. Patient 1 was presented at age 3 years with translocation (DS/Translocation). Patient 2 was presented at age 4 years with trisomy 21 (DS/Trisomy 21). Written informed consent was obtained from the guardian of each participant.

### Cell culture

Human dermal fibroblasts (HDFs) were derived from the skin via operative incision when the patients underwent cardiac surgery. The HDFs were maintained in Dulbecco modified Eagle medium (DMEM) containing 10% fetal bovine serum (FBS), 1% nonessential amino acids, 0.1% β-mercaptoethanol, and 1% penicillin/streptomycin. The iPSCs and hESC-H7 (WiCell Research Institute, Madison, Wisconsin, USA) were maintained on a protein gelatin (Matrigel; BD) with irradiated mouse embryonic fibroblast-conditioned medium (MEF-CM) in DMEM/F12 supplemented with 20% KnockOut Serum Replacement (Invitrogen), 1% nonessential amino acids, 1 m*M *L-glutamine, 0.1% β-mercaptoethanol, and 4 ng/ml basic fibroblast growth factor (bFGF).

### Lentivirus production

The 293T (ATCC) cells were seeded overnight at 2 × 10^6 ^to 2.5 × 10^6 ^cells per 100-mm dish with DMEM supplemented with 10% FBS. The cells were co-transfected with pDM2.G and pSPAX2, along with pLM-vexGFP-OCT4, pLM-mCitrine-SOX2, pLM-mCherry-KLF4, or pLM-mCerulean-c-MYC (Addgene) with calcium phosphate cell transfection, as previously described [26]. Around 48 hours after transfection, the medium containing the lentivirus was collected, and the cellular debris was removed with centrifugation. The supernate was filtered through a 0.45-μm filter, and the lentivirus was pelleted with ultracentrifugation at 33,000 rpm in 45 Ti rotors (Beckman) for 90 minutes at 4°C. The lentivirus particles were resuspended in the medium and stored at -80°C.

### Generation of DS-specific iPSCs

For the reprogramming experiments, the HDFs were infected with a cocktail of lentiviruses expressing exogenous OCT4, SOX2, KLF4, and c-MYC in the presence of 6 μg/ml of polybrene [27]. After 24 hours, the medium was replaced with MEF-CM supplemented with 1 m*M *valproic acid, which lasted for 2 weeks [28]. The medium was changed every other day. On day 30, hESC-like colonies were picked up based on the morphology and plated onto Matrigel-coated wells in MEF-CM.

### Alkaline phosphatase staining and immunostaining

Alkaline phosphatase (AP) staining was performed by using the Alkaline Phosphatase Detection Kit (Millipore) according to the manufacturer's instructions. For the immunostaining, the fixed cells were incubated with the primary antibodies of OCT4 (1:100; Chemicon), SSEA4 (1:100; Chemicon), TRA-1-60 (1:100; Chemicon), TRA-1-81 (1:100; Chemicon), beta-Tubulin III (Tuj1) (1:500; Sigma), AFP (1:100; Santa Cruz), and cTnT (1:200; R&D). The secondary antibodies used were cyanine 2 (Cy2)-conjugated rabbit anti-mouse IgM (1:50; Jackson ImmunoResearch) and rhodamine-conjugated AffiniPure goat anti-mouse IgG (1:200; Santa Cruz). The nuclei were stained with 4',6-diamidino-2-phenylindole (DAPI, Sigma).

### Differentiation *in vitro *

For the embryoid body (EB) formation, iPSCs and hESCs were harvested with treatment with dispase. Cell clumps were cultured in a suspension in ultra-low-attachment plates containing the EB differentiation medium (DMEM supplemented with 20% FBS, 1% nonessential amino acids, and 1% penicillin/streptomycin). After 8 days as a floating culture, the EBs were transferred to a gelatin-coated plate and cultured in the same medium for another 8 days.

### Teratoma formation

For the teratoma formation, 5 × 10^6 ^cells of each iPSC line were harvested and subcutaneously injected into nonobese diabetic-severe combined immunodeficient (NOD-SCID) mice with the Matrigel [29]. Eight weeks after injection, the teratomas were dissected and fixed with PBS containing 4% paraformaldehyde. The paraffin-embedded tissues were sectioned and stained with hematoxylin and eosin (HE staining).

### Semiquantitative and quantitative reverse-transcription polymerase chain reaction (RT-PCR)

Total RNA was extracted from the cells by using TRIzol (Invitrogen) and was transcribed into cDNA by using a HiFi-MMLV cDNA kit (CWBIO). Semiquantitative RT-PCR was subsequently performed to detect the expression of total and endogenous pluripotency-associated genes, as well as the genes representing the three germ layers. Quantitative RT-PCR was performed by using a RealSuper Mixture (with Rox) to indicate the total expression level of the four pluripotency-associated genes. The transcript levels were determined by using the 7500 Real-Time PCR System (Applied Biosystems). The gene expression was normalized to β-actin (ACTB) as the internal standard. The primers are provided in Supplementary Table 2.

### Bisulfite sequencing

Genomic DNA was purified and treated with a CpGenome DNA Modification Kit (Chemicon) according to the manufacturer's recommendations. The treated DNA was subjected to nested PCR to obtain the promoter regions of human OCT4. The primer information is provided in Supplementary Table 2. The PCR products were cloned into pGEM-T Easy plasmids and transformed into TOP10 cells. Ten clones of each sample were sequenced.

### Karyotype analysis and short tandem repeat analysis

Karyotype analysis was conducted by using standard protocols for the chromosomal Giemsa (G)-banding at the Peking University Health Science Center. Short tandem repeat analysis was performed at the Center of Forensic Sciences, Beijing Genomics Institute.

### Statistical analysis

The results are reported as the mean ± SEM. The statistical significance of the differences was determined by a one-way ANOVA. Values with *P *< 0.05 were considered statistically significant.

## Results

### Reprogramming of HDFs from patients with DS into iPSCs

To generate iPSC-DS, HDF-DS were obtained from skin biopsies obtained from the boys with congenital heart disease when the patients underwent cardiac surgery.

**Figure 1:**
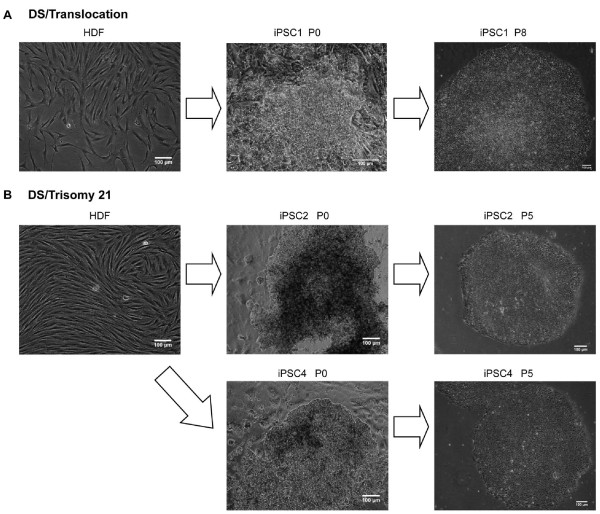
**The reprogramming cells show a similar morphology to hESCs**. **(A) **One induced pluripotent stem cell (iPSC) line derived from a Down syndrome (DS) patient with translocation (DS/translocation). Morphology of the parental human dermal fibroblasts (HDFs; left), iPSC1 (colony no.1 reprogrammed from HDFs-DS/translocation) before picking the colony (middle), and at passage 8 (right). **(B) **Two iPSC lines derived from a DS patient with trisomy 21 (DS/trisomy 21). Morphology of the parental HDFs (left), iPSCs 2 (Colony No. 2 reprogrammed from HDFs-DS/trisomy 21) and iPSC4 (Colony No. 4 reprogrammed from HDFs-DS/trisomy 21) before picking the colonies (middle), and at passage 5 (right). Scale bars, 100 μm.

The lentiviruses containing *OCT4*, *SOX2*, *KLF4*, and *c-MYC *were introduced into HDFs [30], and the cells were plated onto Matrigel-coated plates. On the day after transduction, MEF-CM was applied to the Matrigel-coated plates with 1 m*M *valproic acid, which lasted for 2 weeks [28]. Approximately 7 days after transduction, small cell clumps clearly distinguishable from the fibroblasts started to grow. At around 20 days, some hESC-like colonies with morphologies resembling those of hESCs with a high nuclear-to-cytoplasmic ratio began to form. The colonies were picked out and propagated in MEF-CM on Matrigel. After several passage expansions, several iPSC lines were selected for further analysis (Figure 1).

### Expression of pluripotent-specific markers by human iPSC-DS lines and silencing of the lentivirally delivered transgene

**Figure 2:**
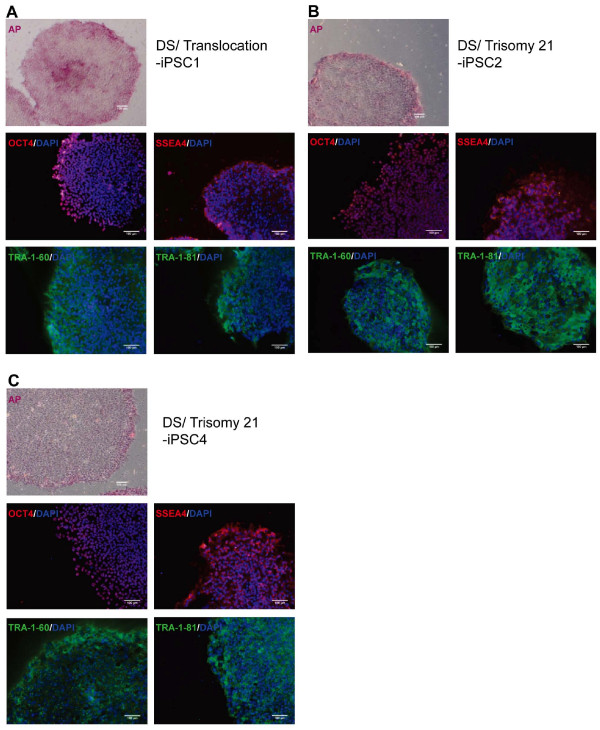
**Human induced pluripotent stem cells (iPSCs-DSs) lines express pluripotency markers**. The iPSCs expressed alkaline phosphatase. As shown by the immunostaining results, the cells expressed pluripotency markers, including OCT4, SSEA4, TRA-1-60, and TRA-1-81. **(A) **DS/translocation-iPSC1. **(B) **DS/trisomy 21-iPSC2. **(C) **DS/trisomy 21-iPSC4. Nuclei are stained with DAPI (blue). Scale bars, 100 μm.

**Figure 3:**
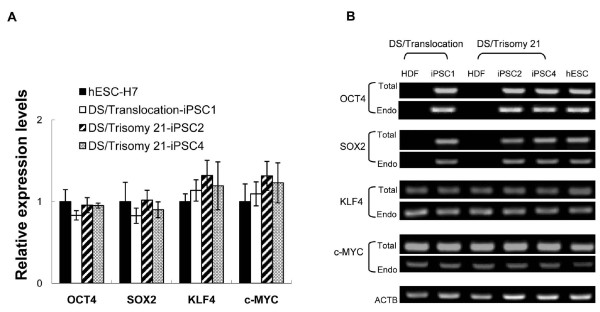
**The induced pluripotent stem cells (iPSCs-DSs) express pluripotency-associated genes**. **(A) **The total gene-expression levels of OCT4, SOX2, KLF4, and c-MYC were determined by using quantitative reverse transcription polymerase chain reaction (RT-PCR) in iPSCs-DS and hESCs. The differences in the expression levels of H7 hESCs and iPSCs-DS were not significant. The results are reported as mean ± SEM (*n *= 3). **(B) **The endogenous (Endo) and total (Total) expression levels of OCT4, SOX2, KLF4, and c-MYC were analyzed with semiquantitative RT-PCR and compared with parental human dermal fibroblasts. β-Actin (ACTB) was used as the positive control.

To characterize the generated iPSC-DSs, their expression of the pluripotent markers was examined by using alkaline phosphatase (AP) staining and immunostaining. The reprogrammed cells were positive for AP activity and expressed the pluripotent-specific surface antigens SSEA4, TRA-1-60, and TRA-1-81, and the nuclear transcription factor OCT4 (Figure 2). Moreover, quantitative RT-PCR results show the total expression of the pluripotency-associated genes, including *OCT4*, *SOX2*, *KLF4*, and *c-MYC *(Figure 3A). The expression of *OCT4*, *SOX2*, *KLF4*, and *c-MYC *in the iPSC lines was similar to that of the hESCs. In addition, the endogenous expression of *OCT4*, *SOX2*, *KLF4*, and *c-MYC *was analyzed with semiquantitative RT-PCR (Figure 3B). The expression of the endogenous *OCT4 *and *SOX2 *was consistently upregulated relative to the parental fibroblasts, and *KLF4 *and *c-MYC *were expressed in iPSCs and hESCs, as well as in the fibroblasts. As previously described [30], a pronounced lentiviral vector silencing is a characteristic of successfully reprogrammed iPSC clones. In the present study, the lentiviral vectors encoding the four reprogramming factors co-expressed with discernable fluorescent proteins were used to generate the iPSCs-DS. Monitoring the expression of each fluorescent protein is determined to reveal the expression of individual reprogramming factor. The expression of four fluorescent proteins that represent the transgenic expression was predominantly silenced in all of the iPSC-DS lines (data not shown). The results demonstrate the successfully reprogrammed iPSC-DS.

### Activation of the promoters of hESC-specific genes in human iPSCs

**Figure 4:**
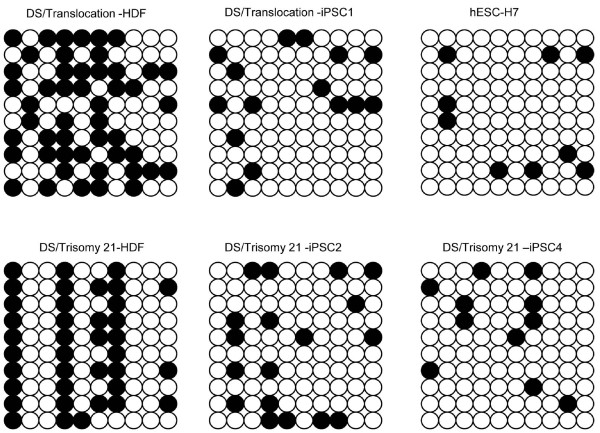
**The induced pluripotent stem cells (iPSCs-DSs) have a methylation status similar to that of hESCs**. The methylation status of the endogenous OCT4 promoter in iPSCs-DS and parental human dermal fibroblasts was analyzed with bisulfite sequencing. Open circles indicate unmethylated CpGs, and solid circles indicate methylated CpGs.

The DNA-methylation status of genomic DNA from iPSC-DS, parental HDFs, and H7 hESCs were analyzed to assess the epigenetic reprogramming of iPSC-DS [31]. Among the four transcription factors, *OCT4 *plays a key role in maintaining and regaining stem cell pluripotency [32], and the promoter regions of *OCT4 *were subjected to bisulfite genomic sequencing. The results demonstrate the demethylation status of cytosine guanine dinucleotides (CpG) in the promoter region of *OCT4 *for the iPSCs and their parental fibroblasts (Figure 4). The *OCT4 *promoter was released and activated in the reprogrammed iPSC-DSs, compared with that in the parental fibroblasts, and was found to be similar to that of hESCs. These results are consistent with the occurrence of epigenetic remodeling during reprogramming through gene transduction [11,12].

### Embryoid body-mediated differentiation of iPSC-DS *in vitro *

**Figure 5:**
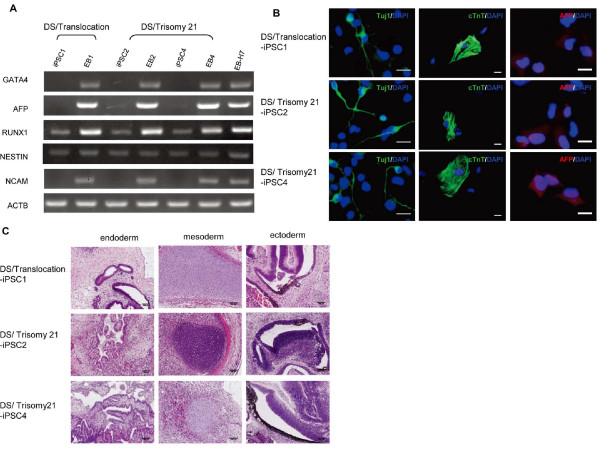
**The induced pluripotent stem cells (iPSCs-DSs) maintain pluripotency *in vitro *and *in vivo***. **(A) **Embryoid bodies (EBs) were formed by the iPSCs-DS and semiquantitative reverse transcription polymerase chain reaction (RT-PCR) analyses of lineage markers that represent the three germ layers. **(B) **Immunostaining showing expression of the three lineages markers Tuj1 (ectoderm), cTnT (mesoderm), and α-fetoprotein (endoderm) in DS-iPSC clones from three independent iPSC derivations subjected to EBs differentiation. **(C) **Teratomas were collected and stained with hematoxylin and eosin. The tissues originating from the three embryonic germ layers were present in the teratomas. Scale bars, 100 μm.

To evaluate the differentiation ability of iPSC-DS *in vitro*, EBs were formed in suspension cultivation. After 8 days in a suspension culture, the EBs were transferred onto the gelatin-coated plates, and the cultivation was continued for another 8 days. The attached cells were then collected for semiquantitative RT-PCR to detect the expression of GATA4 (endoderm), AFP (endoderm), RUNX1 (mesoderm), NESTIN (ectoderm), and NCAM (ectoderm). Figure 5A shows that iPSC-DS cells possess the ability to differentiate into cell types originating from all three germ layers *in vitro*. Also, immunostaining analyses of lineage markers represent the three germ layers (Figure 5B).

### Teratoma formation from human iPSCs

Ultimately, the fully reprogrammed cells should be able to form teratomas containing tissue cell types of all three germ layers *in vivo *to be truly considered hESC-like iPSCs. The iPSC-DS cells were injected into NOD-SCID mice to observe the teratoma formation. Eight weeks after injection, the teratomas were harvested for HE staining. The teratomas contained derivatives of the endoderm (glandular structures), mesoderm (cartilage), and ectoderm (pigmented epithelium and melanocytes) (Figure 5C). These results suggest that iPSC-DS can spontaneously differentiate into derivatives of all three germ layers *in vivo*. 

### Human iPSC-DS derived from HDF-DS, not from a contamination of laboratory hESCs

**Figure 6:**
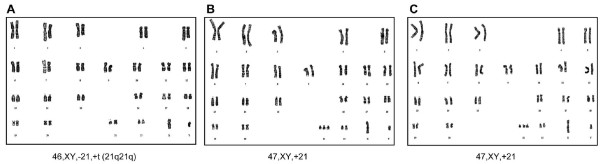
**The induced pluripotent stem cells (iPSCs-DSs) maintain the same karyotypes as the parental human dermal fibroblasts under G-band analysis**. **(A) **DS/translocation-iPSC1 at passage 15. **(B) **DS/trisomy 21-iPSC2 at passage 12. **(C) **DS/trisomy 21-iPSC4 at passage 12.

Short tandem repeat analysis was conducted to confirm the origin of the human iPSC-DS. The patterns of 18 short tandem repeats between iPSCs and parental HDFs completely matched (Supplementary Table 1). These patterns differed from those of the hESCs cultured in our laboratory. Karyotype analysis confirms that each iPSC line generated from the parental fibroblasts maintained the normal euploid karyotype after reprogramming (Figure 6).

## Discussion

DS is caused by the trisomy of part or all of human chromosome 21, but its phenotypic features differ from person to person. Understanding and investigating the DS mechanisms are challenges because of the genetic complexity and individual variability of DS phenotypes. Mouse models are useful for DS research, but DS modeling in mice is particularly complex because of the size of the region involved as well as the number of candidate genes and incomplete synteny between animal and human chromosomes. The hESCs [33], which have the potential to provide an unlimited supply of different cell types for tissue replacement, drug screening, and functional genomics applications, were first derived by Thomson *et al. *[34]. Although some hESCs with trisomy 21 have been established, the production of different types for DS studies is also limited because of ethical challenges. In addition, the transplantation of hESC-differentiated cells can trigger immune-rejection by the host. In the present study, reprogramming iPSC-DS could provide a source for DS modeling, optimal patient care, drug screening, and eventually autologous cell-replacement therapies. Compared with a previous study by Park *et al. *[15], we generated iPSCs from DS patients with various karyotypes (trisomy 21 and translocation). We did not find significant differences between iPSC-DS/trisomy 21 and iPSC-DS/translocation cell lines in the abilities of self-renewal and pluripotency. Although our results were consistent with the previous report, the current study confirmed that iPSCs can be generated from patients with various karyotypes.

iPSCs were first derived by Yamnaka *et al. *[11] from mouse fibroblasts with retroviral vectors. In our study, we used lentiviral vectors encoding the four transcription factors (OCT4, SOX2, KLF4, and c-MYC) co-expressed with discernable fluorescent proteins to generate the iPSC-DS. An obvious difference between retroviral and lentiviral vectors is the degree of silencing to which they are subject in pluripotent cells [35]. We have determined the silencing of exogenous genes by monitoring expression of each fluorescent protein. It is know that integration of retroviral and lentiviral vectors into the genome may affect the behaviors of the iPSCs, for sustained expression or reaction of reprogramming factors to prevent proper differentiation of iPSCs. Even for future clinical applications, it would be necessary to reprogram patient cells by using nonintegrating vectors [31,36]. A number of studies have described successful derivation of iPSCs by using adenoviral vectors, episomal vectors, or the introduction of reprogramming proteins, synthetic modified mRNA [37-40], but a vector-free protocol is usually limited by the low efficiency of iPSCs derivation. Given the rapid pace of the field, further optimization of a highly efficient and vector-free method of generation of iPSCs will facilitate the clinical translation of this technology.

The iPSC technology allows the generation of disease- and patient-specific iPSCs and offers a new cell model for human disease-mechanism studies without ethical controversies, as well as transplantation medicines without immune rejection. With the technology, one group created a human Marfan syndrome "model" to prove the hypothesis that fibrillin-1 mutations result in the disease phenotypes [41]. The hypothesis that the DS gene is dosage dependent predicts that a region critical for specific phenotypes contains a dosage-sensitive gene or genes [1], of which a dosage imbalance results in the formation of different phenotypes. The establishment of different types of iPSC-DSs can be used to test this hypothesis.

DS is the major cause of congenital heart disease and the most frequent genetic cause of mental retardation. It is also associated with increased risks of leukemia and immune system defects. In a future study, the iPSC-DS "model" can be used to differentiate cardiomyocytes, neuron cells, and lymphocytes for transplantation to improve the phenotypes of patients. For example, ventricular septal defect is a common congenital heart disorder. In future clinical applications, it may be possible to use functional cardiac tissue directly differentiated from autologous iPSC in three-dimensional scaffold to repair the defect [42]. In addition, the study of the differences of cell behavior between the iPSCs-DS, iPSCs derived from normal patients, and iPSCs-DS from different karyotypes would be helpful to understand the disease mechanism and the development of personalized therapy.

## Conclusions

Human dermal fibroblasts from patients with DS of various karyotypes were successfully reprogrammed into iPSCs. All the iPSC-DS retained the abilities of self-renewal and pluripotent potentials *in vitro *and *in vivo*. DS-derived iPSCs offer the possibility for DS cell modeling, optimal patient care, drug discovery, and eventually, autologous cell-replacement therapies.

## Abbreviations

AP: alkaline phosphatase; DS: Down syndrome; EB: embryoid body; HDFs: human dermal fibroblasts; HE: hematoxylin and eosin; hESCs: human embryonic stem cells; iPSCs: induced pluripotent stem cells; MEF-CM: mouse embryonic fibroblast-conditioned medium; NOD-SCID: nonobese diabetic-severe combined immunodeficient mice; RT-PCR: reverse-transcription polymerase chain reaction.

## Competing interests

The authors declare that they have no competing interests.

## Authors' contributions

XNM and YBW carried out the experiments for the generation of iPSC-DS, tested the expression of pluripotency-associated genes by immunostaining or semiquantitative and quantitative RT-PCR, and the pluripotency of the iPSC *in vitro *and *in vivo*. XNM and YBW also participated in analyzing the data and drafted the manuscript. HHC derived the HDFs from the patients and prepared the MEFs for the experiments with QZM. QHW, CCS, and SSH gave some advice for this manuscript. HZ and YM designed the study and reviewed and revised the manuscript. All the authors read and approved the manuscript for publication.

## Supplementary Material

Additional file 1 **Supplementary Table 1**. 18 short tandem repeats profiles of parental HDFs, iPSCs-DS, and hESCs-H7. Click here for file

Additional file 2 **Supplementary Table 2**. Primers used for PCR, RT-PCR, and quantitative RT-PCR. Click here for file
