# Supplementary material for: Generation of disease-specific induced pluripotent stem cells from patients with different karyotypes of Down syndrome
Source: Stem Cell Res Ther. 2012 Apr 18;3(2):14. doi: 10.1186/scrt105 (PMC3392774; doi:10.1186/scrt105)
Supplement: Additional file 1 — Supplementary Table 1. 18 short tandem repeats profiles of parental HDFs, iPSCs-DS, and hESCs-H7. [file scrt105-S1.DOC]

**Table S1. 18 short tandem repeats profiles of parental HDFs, iPSCs-DS, and hESCs-H7.**

| Locus/sample | DS/Translocation -HDF | DS/Translocation -iPSC1 | DS/Trisomy 21 -HDF | DS/ Trisomy21 -iPSC2 | DS/Trisomy 21 -iPSC4 | hESC-H7 |
| --- | --- | --- | --- | --- | --- | --- |
| D3S1358 | 15,15 | 15,15 | 15,15 | 15,15 | 15,15 | 15,16 |
| D13S317 | 9,11 | 9,11 | 9,12 | 9,12 | 9,12 | 11,12 |
| D7S820 | 11,11 | 11,11 | 11,12 | 11,12 | 11,12 | 10,11 |
| D16S539 | 9,11 | 9,11 | 9,10 | 9,10 | 9,10 | 12,13 |
| Penta E | 14,14 | 14,14 | 15,18 | 15,18 | 15,18 | 11,13 |
| TPOX | 8,8 | 8,8 | 9,11 | 9,11 | 9,11 | 8,11 |
| TH01 | 9,9.3 | 9,9.3 | 9,9 | 9,9 | 9,9 | 6,6 |
| D2S1338 | 18,24 | 18,24 | 19,24 | 19,24 | 19,24 | 20,24 |
| CSF1PO | 10,11 | 10,11 | 12,12 | 12,12 | 12,12 | 12,12 |
| D19S433 | 14,14.2 | 14,14.2 | 14,15.2 | 14,15.2 | 14,15.2 | 13,14.2 |
| vWA | 17,17 | 17,17 | 14,15 | 14,15 | 14,15 | 14,15 |
| D5S818 | 12,13 | 12,13 | 10,12 | 10,12 | 10,12 | 11,13 |
| FGA | 23.2,25 | 23.2,25 | 23,24 | 23,24 | 23,24 | 21,22 |
| D6S1043 | 11,17 | 11,17 | 12,15 | 12,15 | 12,15 | 18,18 |
| D8S1179 | 11,13 | 11,13 | 12,12 | 12,12 | 12,12 | 13,14 |
| D21S11 | 29,30 | 29,30 | 29,30 | 29,30 | 29,30 | 30,31.2 |
| D18S51 | 14,15 | 14,15 | 17,22 | 17,22 | 17,22 | 12,15 |
| AMEL | X,Y | X,Y | X,Y | X,Y | X,Y | X,X |
