# Supplementary material for: Generation of disease-specific induced pluripotent stem cells from patients with different karyotypes of Down syndrome
Source: Stem Cell Res Ther. 2012 Apr 18;3(2):14. doi: 10.1186/scrt105 (PMC3392774; doi:10.1186/scrt105)
Supplement: Additional file 2 — Supplementary Table 2. Primers used for PCR, RT-PCR, and quantitative RT-PCR. [file scrt105-S2.DOC]

**Table S2. Primers used for PCR, RT-PCR and quantitative RT-PCR.**

| **primers** | **Forward sequence** | **Reverse sequence** |
| --- | --- | --- |
| **For quantitative RT-PCR** | | |
| OCT4 | AGCGAACCAGTATCGAGAAAC | TTACAGAACCACACTCGGAC |
| SOX2 | AGCTACAGCATGATGCAGGA | GGTCATGGAGTTGTACTGCA |
| KLF4 | TCTCAAGGCACACCTGCGAA | TAGTGCCTGGTCAGTTCATC |
| c-MYC | ACTCTGAGGAGGAACAAGAA | TAGTGCCTGGTCAGTTCATC |
| ACTB | TGAAGTGTGACGTGGACATC | GGAGGAGCAATGATCTTGAT |
| **For RT-PCR** | | |
| OCT4-Total | AGCGAACCAGTATCGAGAAC | TTACAGAACCACACTCGGAC |
| OCT4-Endo | CCTCACTTCACTGCACTGTA | CAGGTTTTCTTTCCCTAGCT |
| SOX2-Total | AGCTACAGCATGATGCAGGA | GGTCATGGAGTTGTACTGCA |
| SOX2-Endo | AGCTACAGCATGATGCAGGA | GGTCATGGAGTTGTACTGCA |
| KLF4-Total | TCTCAAGGCACACCTGCGAA | TAGTGCCTGGTCAGTTCATC |
| KLF4-Endo | GATGAACTGACCAGGCACTA | GTGGGTCATATCCACTGTCT |
| c-MYC-Total | ACTCTGAGGAGGAACAAGAA | TGGAGACGTGGCACCTCTT |
| c-MYC-Endo | TGCCTCAAATTGGACTTTGG | GATTGAAATTCTGTGTAACTGC |
| ACTB | TGAAGTGTGACGTGGACATC | GGAGGAGCAATGATCTTGAT |
| GATA4 | CTAGACCGTGGGTTTTGCAT | TGGGTTAAGTGCCCCTGTAG |
| AFP | AGCTTGGTGGTGGATGAAAC | CCCTCTTCAGCAAAGCAGAC |
| RUNX1 | CCCTAGGGGATGTTCCAGAT | TGAAGCTTTTCCCTCTTCCA |
| NCAM | ATGGAAACTCTATTAAAGTGAACCTG | TAGACCTCATACTCAGCATTCCAGT |
| NESTIN | GCGTTGGAACAGAGGTTGGA | TGGGAGCAAAGATCCAAGAC |
| **For bisulfite-sequencing PCR** | | |
| Oct4-meth-1 | TTATTGTTATTATTATTAGGTAAATATTT | AAAATCCCCCACACCTCAAAACCTAACC |
| Oct4-meth-2 | GGGGTTAGAGGTTAAGGTTAGTGGGTG | AAACCTTAAAAACTTAACCAAATC |

**Reference**

1. Park IH, Arora N, Huo H, Maherali N, Ahfeldt T, Shimamura A, Lensch MW, Cowan C, Hochedlinger K, Daley GQ: **Disease-specific induced pluripotent stem cells.** *Cell* 2008, **134:**877-886.

2. Li W, Wang X, Fan W, Zhao P, Chan YC, Chen S, Zhang S, Guo X, Zhang Y, Li Y, Cai J, Qin D, Li X, Yang J, Peng T, Zychlinski D, Hoffmann D, Zhang R, Deng K, Ng KM, Menten B, Zhong M, Wu J, Li Z, Chen Y, Schambach A, Tse HF, Pei D, Esteban MA: **Modeling abnormal early development with induced pluripotent stem cells from aneuploid syndromes.** *Hum Mol Genet* 2012, **21:**32-45.
